# Optimizing protein tokenization: reduced amino acid alphabets for efficient and accurate protein language models

**DOI:** 10.1093/bioinformatics/btag226

**Published:** 2026-07-07

**Authors:** Ella Rannon, David Burstein

**Affiliations:** The Shmunis School of Biomedicine and Cancer research, George S. Wise Faculty of Life Sciences, Tel Aviv University, Tel Aviv, 6997801, Israel; The Shmunis School of Biomedicine and Cancer research, George S. Wise Faculty of Life Sciences, Tel Aviv University, Tel Aviv, 6997801, Israel

## Abstract

**Motivation:**

Protein language models (pLMs) typically tokenize sequences at the single-amino-acid level using a 20-residue alphabet, resulting in long input sequences and high computational cost. Sub-word tokenization methods such as Byte Pair Encoding (BPE) can reduce sequence length but are limited by the sparsity of long patterns in proteins encoded by the standard amino acid alphabet. Reduced amino acid alphabets, which group residues by physicochemical properties, offer a potential solution but their performances with sub-word tokenization have not been systematically studied.

**Results:**

We investigate the combined use of reduced amino acid alphabets and BPE tokenization in protein language models. We pre-train RoBERTa-based pLMs *de novo* using multiple reduced alphabets and evaluate them across diverse downstream tasks. Our results show that reduced alphabets enable substantially shorter input sequences and faster training and inference. These findings suggest that alphabet reduction may facilitate more effective sub-word tokenization, enabling increased efficiency with marginal impact on predictive performance, and for specific tasks even improving accuracy.

**Availability and implementation:**

Models, tokenizers, and code are available at github.com/burstein-lab/BioTokenizers.

## 1 Introduction

Advances in high-throughput sequencing have generated vast amounts of genomic sequence data, much of which remains unannotated (Sayers et al. 2025). The ability of language models (LMs) to learn complex patterns from large unlabeled corpora makes them well-suited to address this challenge (Bepler and Berger 2021, Iuchi et al. 2021, Ofer et al. 2021, Zhang et al. 2023, Oikonomou et al. 2024, Rannon and Burstein 2025). In particular, protein language models (pLMs) have demonstrated strong performance in capturing evolutionary, structural, and functional signals directly from amino acid sequences (Alley et al. 2019, Rao et al. 2019, Rives et al. 2021, Elnaggar et al. 2022, Lin et al. 2023).

A key design choice in pLMs is how protein sequences are segmented to basic language units called tokens (Testagrose and Boucher 2025). Many pLMs adopt character-level tokenization, treating individual amino acids as tokens (Alley et al. 2019, Heinzinger et al. 2019, Rao et al. 2019, Cao and Shen 2021, Rives et al. 2021, Lin et al. 2023). While this approach enables position-specific predictions and maintains a small vocabulary, it results in tokens encoding limited contextual information and long input sequences, leading to increased computational cost. Fixed-length *k*-mer tokenization (Asgari and Mofrad 2015, Kimothi et al. 2016) partially addresses this limitation but introduces sensitivity to insertions and deletions, severe vocabulary sparsity when utilizing a large k, and is based on the unrealistic assumption that informative sequence elements share a fixed length.

Sub-word tokenization methods, such as Byte Pair Encoding (BPE) (Sennrich et al. 2016), provide a flexible alternative by learning variable-length tokens from frequently co-occurring amino acid patterns. By treating protein sequences as raw symbol streams without predefined word boundaries, these methods are well-suited to biological sequences lacking linguistic structure. The resulting variable-length tokens can capture higher-order sequence features, including recurrent motifs of biological relevance, while reducing vocabulary sparsity and naturally handling unseen tokens. Sub-word tokenization has been successfully applied in several pLMs (Asgari et al. 2019, Wang et al. 2019, Elnaggar et al. 2022, Ferruz et al. 2022) and evaluated across vocabulary sizes (Suyunu et al. 2024) and downstream tasks (Dotan et al. 2024, Tan et al. 2024). Nevertheless, its effectiveness is constrained by the sparsity of long patterns in the standard 20-amino-acid alphabet.

Protein sequences can be simplified by using reduced alphabets, where each character represents a group of amino acids with shared physicochemical, functional, or structural properties (Liang et al. 2022). Reducing the amino acid alphabet can increase the frequency of longer recurring patterns with biological significance, enabling BPE to construct longer and more informative tokens. While reduced amino acid alphabets have been previously used in bioinformatics (Murphy et al. 2000, Weathers et al. 2004, Peterson et al. 2009, Ofer and Linial 2015), prior evaluations of these alphabets in pLMs focused exclusively on single-residue tokenization and reported reduced model performance (Ieremie et al. 2024). The trade-off between alphabet resolution and token length has not yet been systematically explored on biological data. Combining reduced alphabets with sub-word tokenization may enable the construction of longer tokens while preserving or even enhancing the model’s ability to capture relevant biological patterns.

Here, we investigated the combined use of reduced amino acid alphabets and BPE sub-word tokenization in protein language models. We pre-trained pLMs from scratch using different sizes of reduced alphabets and evaluated their performance across diverse downstream tasks. These include classification tasks for solubility, enzymes, transporter identification, two-component systems, protein-protein interactions (PPIs), as well as regression tasks for fluorescence, optimal temperature, and stability regression. We showed that reduced alphabets improved model performance on some tasks, while substantially reducing input sequence length and runtime. Our results highlight that incorporating prior knowledge of amino acid properties through alphabet reduction is an effective strategy for improving both the efficiency and, in certain tasks, the performance of protein language models.

## 2 Methods

### 2.1 Datasets

#### 2.1.1 Corpus

This study’s corpus comprised protein sequences from assembled metagenomic contigs across various ecosystems from EBI’s Mgnify (Mitchell et al. 2020), together with proteins from assembled genomes and metagenomes in the NCBI GenBank Whole Genome Sequencing (WGS) database (Sayers et al. 2020), after filtering out Fungi, Metazoan, and Viridiplantae (downloaded on March 14, 2020). CD-HIT (Fu et al. 2012) (v4.6; -g 1 -s 0.8 -c 0.8) was utilized to reduce redundancy, retaining only the representative sequences from each cluster. The corpus was then split into test and training sets as follows. Genomes of isolated bacteria were assigned either to the training or test set, enforcing an 80:20 protein ratio. This ensured that proteins from the same genome do not appear in both the training and test sets. For metagenomes, samples from each ecosystem type were randomly split to reach an 80:20 protein ratio between the training and test sets within each ecosystem type.

#### 2.1.2 Tokenizer training

For each alphabet, a BPE tokenizer was trained on a set of 28.3 million proteins from the corpus training set. Specifically, it included approximately 100 000 proteins randomly sampled from each ecosystem type in the corpus training set, as well as proteins from RefSeq’s representative and reference genomes, resulting in a total of 28 300 268 proteins. Vocabulary properties were assessed for each tokenizer on an evaluation set comprising ∼25 000 proteins per ecosystem type from the corpus test set, yielding 8 650 002 proteins.

#### 2.1.3 Pre-training

Using the trained BPE tokenizer, a RoBERTa-based protein language model was pre-trained. The training set included a random subset of 15 million proteins from the corpus training set.

#### 2.1.4 Homology detection

For this task, all protein sequences were obtained from UniRef90 (Suzek et al. 2015) and family annotations from InterPro (Paysan-Lafosse et al. 2023) (downloaded on August 3, 2025). The smallest 10% of families were removed. For each remaining family, 15 protein pairs were randomly sampled as positive examples. An approximately equal number of negative pairs was generated by sampling proteins from different families, ensuring each family appeared in 30 total pairs. The final dataset comprised 663 900 protein pairs.

To minimize potential data leakage between training and test sets, any test protein sharing more than 50% sequence identity at 80% or greater coverage with any training protein was removed using Linclust (Steinegger and Söding 2018) (—min-seq-id 0.5 -c 0.8; commit bad16c76).

#### 2.1.5 Solubility and signal peptide prediction

The solubility dataset was acquired from DeepLocPro (Moreno et al. 2024) (v1.0), and contained 9915 training and 1991 test proteins after applying the 50% identity filtering to avoid potential leakage, as described above. The data was split into training and test in an 80:20 ratio using the original cross-validation partitions, with folds 0–3 taken as train, and fold 4 as test. The signal peptide training and test sets were obtained from SignalP (Teufel et al. 2022) (v6.0), resulting in 20 290 training and 2021 test proteins after the same 50% identity filtering. In both cases, only a binary label was used.

#### 2.1.6 Enzyme, transporter, and two-component system prediction

Kyoto Encyclopedia of Genes and Genomes (KEGG) (Kanehisa and Goto 2000) orthology (KO) identifiers were assigned to our corpus using the mapping from Miller et al. (2022). Briefly, proteins associated with the same KO were subclustered using MMseqs2 cluster -s 7.5 -c 0.5 (Steinegger and Söding 2017), and each cluster was aligned with MAFFT (Katoh et al. 2002) and used to construct an HMM profile (Eddy 2011) for corpus annotation (Miller et al. 2022). Positive labels were assigned from the KEGG BRITE hierarchies representing enzymes, transporters, and two-component systems (ko01000, ko02000, and ko02022, respectively). Negative examples consisted of proteins annotated with KOs not belonging to the corresponding BRITE hierarchy. KOs were split into training, validation, and test sets in an 8:1:1 ratio based on their frequency in the data. Negative proteins were sampled to achieve a 10:1 negative-to-positive ratio. Due to the large resulting dataset size, stratified subsampling was applied, retaining 25% of the data for enzyme and transporter prediction tasks, and 30% for the two-component system prediction task. Finally, test proteins sharing ≥50% identity with any training protein were filtered out as described above. This process resulted in 4 235 473, 4 190 082, and 1 356 528 train proteins; 510 790, 497 336, and 208 524 validation proteins; and 505 100, 508 093, and 215 647 test proteins, for the enzyme, transporter, and two-component system prediction tasks, respectively.

#### 2.1.7 PPI prediction

The dataset was based on B-PPI-DB (Agassy et al. 2025), including the *H. pylori* protein pairs used to test B-PPI (Agassy et al. 2025), which were processed as the other pairs in B-PPI-DB. The final PPI database included 162 703 training, 20 338 validation, and 20 338 test protein pairs. As this dataset construction process included Linclust clustering (—min-seq-id 0.4 -c 0.8), no further filtering was required.

#### 2.1.8 Stability and fluorescence prediction

The datasets were obtained from the TAPE benchmark (Rao et al. 2019) (downloaded on September 16, 2025). The stability and fluorescence datasets included 53 614 and 21 446 training proteins, 2512 and 5362 validation proteins, and 12 851 and 27 217 test proteins, respectively. The stability test set was reduced to 728 proteins after the 50% identity filtering. The fluorescence dataset was specifically included to assess model performance on closely related sequence variants. Since this dataset comprised variants of a single parent GFP sequence differing by only a few amino acids, the 50% identity filtering was not applied.

#### 2.1.9 Optimal temperature prediction

The dataset was downloaded from BRENDA (Hauenstein et al. 2025) (2025.1 release, version 1). Proteins with missing labels (“−999”) were removed. Sequences were clustered using MMseqs2 (Steinegger and Söding 2017) (-s 7.5 -c 0.8; commit bad16c76). Proteins labeled with a temperature range rather than a single value were removed, and the remaining proteins in their clusters were assigned to the test set. The sequences of all other clusters were assigned to the training set. This resulted in 6991 train proteins and 2591 test proteins after the 50% identity filtering of test proteins, as described above.

### 2.2 Tokenizer and model training

We evaluated five reduced amino acid alphabets of varying sizes based on different residue properties (Table 1). For each alphabet, a BPE tokenizer (Sennrich, Haddow and Birch 2016) was trained on the tokenizer dataset using a minimum token frequency of 2 and a vocabulary size of 5,000. This size was chosen to enable meaningful differences in token lengths across alphabets while remaining computationally tractable, as smaller vocabularies would yield uniformly short tokens and obscure the effects of alphabet reduction. The same vocabulary size was used across all alphabets to ensure a controlled comparison, allocating an equal “representational budget” and isolating the alphabet as the primary variable. A corresponding RoBERTa-based model (Liu et al. 2019) (termed ProtBERTa_<alphabet size>) was then pre-trained, consisting of 12 attention heads, eight hidden layers, and a hidden dimension of 768. Each model was pre-trained on a single NVIDIA RTX A6000 GPU for five epochs with a batch size of 64, gradient accumulation of 8, and a maximum sequence length of 1026 tokens, comprised of 1024 sequence tokens and two special tokens: <s> and </s> for marking the beginning and the end of the sequence, respectively. Pre-training was carried out using the masked language modeling (MLM) objective with a masking probability of 0.15.

**Table 1:** The amino acid alphabets used in this study and the groups of amino acids represented in their alphabet. The letter representing each group is written in parentheses.

Alphabet type	Size	Amino acid groups
Amino acids (Baseline)	20	A, C, D, E, F, G, H, I, K, L, M, N, P, Q, R, S, T, V, W, Y
Linclust’s Alphabet	12	AST (A), ND (N), EQ (E), FY (F), IV (I), KR (K), LM (L), C, G, H, P, W
Functional groups	8	GVALI (S, Simple), ST (H, Hydroxy), CM (F, sulfur-containing), FY (M, aromatic), WHP (C, heterocyclic), NQ (N, amine-containing), DE (A, acidic), KR (B, basic)
Polarity	4	GAVLIFWMP (N, non-polar), STCYNQ (P, polar neutral), DE (S, negative charge), HKR (H, positive charge)
Hydrophilic-hydrophobic	2	STNKEQHDRZB (L, hydrophilic), AGILMVPFWCY (B, hydrophobic)

The pre-trained models were adapted on multiple downstream classification and regression tasks. For classification, a task-specific classification head was added, consisting of a dense layer with tanh activation applied to the final hidden state of the class token (<s>), followed by a linear projection layer. Models were trained using the AdamW optimizer, cross-entropy loss with a learning rate of 5⋅10^−5^ and a batch size 64, with gradient accumulation of 2. The models were trained for ten epochs, except for the PPI task, which was trained for two epochs.

For regression tasks, the output hidden states of the base model were mean-pooled and fed to a classification head, composed of a 256-dimensional linear layer with SiLU activation (Elfwing et al. 2017), dropout with a rate of 0.15, and a projection layer. Models were trained using MAE loss with a learning rate of 2⋅10^−5^, weight decay of 0.01, warm-up ratio of 0.1, and a batch size of 64 for 15 epochs.

For all tasks except PPI prediction, the encoder weights were frozen to assess representation quality. In the PPI task, inputs were formatted as <s> protein1_tokens </s> </s> protein2_tokens </s>, which differs from the pre-training setup and therefore, all model parameters were fine-tuned.

### 2.3 Model evaluation

The pre-trained embeddings from each ProtBERTa model were evaluated on the Diverse Genomic Embedding Benchmark (DGEB) (West-Roberts et al. 2024). Following the benchmark protocol, embeddings from the models’ middle (layer 4) and final (layer 7) layers were assessed, and the better-performing layer was reported for each task. The DGEB score was computed as the mean performance across tasks using their pre-defined primary metrics.

We further evaluated the quality of the models’ embedding using two tasks. For homology prediction, cosine similarity was calculated on mean-pooled embeddings for zero-shot classification of protein pairs as homologs. For signal peptide prediction, a kNN classifier (*k *= 5) based on cosine similarity between pooled embeddings was used, with classification scores defined by the proportion of positive neighbors.

Regression models were evaluated using mean squared error (MSE), mean absolute error (MAE), and root mean square error (RMSE), while binary classification models were evaluated using Area Under the ROC (AUROC), Area Under the Precision-Recall curve (AUPR), and maximal F1. For the two-component system prediction task, metrics were computed as macro averages across classes. Additionally, statistical significance between ProtBERTa_20 and each alternative model was assessed using McNemar’s test based on F1-optimizing predictions (or the class with maximal score for multiclass tasks).

Standard error was calculated for each metric using bootstrapping on the test set with n=1000 for all tasks, except for the homology, enzyme, transporter, and two-component system prediction tasks, where n=100 was used instead due to the large size of the datasets.

### 2.4 Runtime evaluation

Training and inference times were measured on a single NVIDIA RTX A6000 GPU. For each task, the test set was randomly subsampled ten times at multiple dataset sizes (increments of 1, 5, 10, 25, and 50), and inference runtime was recorded for the models’ inference on batches of 128 sequences. Prior to measurement, models were warmed up using 100 batches of size 128. For each dataset size, mean runtime and standard error were computed across the repeats. The dataset sizes of most tasks were scaled down by a factor of 1,000, except for the solubility/optimal temperature and stability prediction tasks, which were scaled down by factors of 39 and 500, respectively. Tokenizer training times and tokenization time were measured on a single process on an Intel Xeon Gold 6130 CPU. The tokenizer evaluation set was randomly subsampled ten times at multiple dataset sizes (5000, 10 000, 25 000, 50 000, 75 000, and 100 000).

## 3 Results

### 3.1 Tokenizer training

In this study, we investigated the merits and trade-offs of five different amino acid representations with different abstraction levels for protein language models (Table 1). Specifically, we explored:

1. The traditional 20-letter alphabet representing the 20 different amino acids.
2. A 12-letter representation based on the alphabet developed for the Linclust algorithm for fast clustering of proteins (Steinegger and Söding 2018).
3. An eight-letter representation based on the functional groups of the amino acids (Jain, Jain and Jain 2014).
4. A four-letter representation based on amino acids polarity (Ball, Hill and Scott 2014).
5. A two-letter representation where amino acids are split according to whether they are considered hydrophilic or hydrophobic.

**Figure 1:**
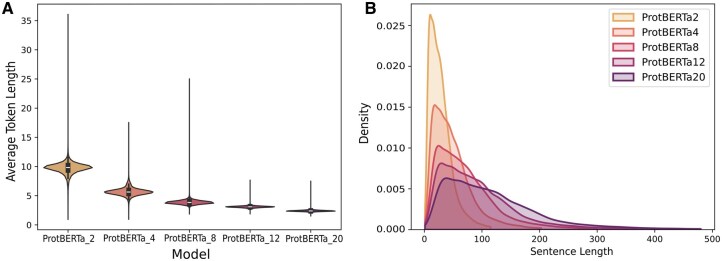
Properties of the BPE tokenizer trained on each amino acid alphabet. (A) Average token length distribution for individual proteins. (B) Distribution of tokenized sentence lengths (top 1% omitted for clarity).

**Figure 2:**
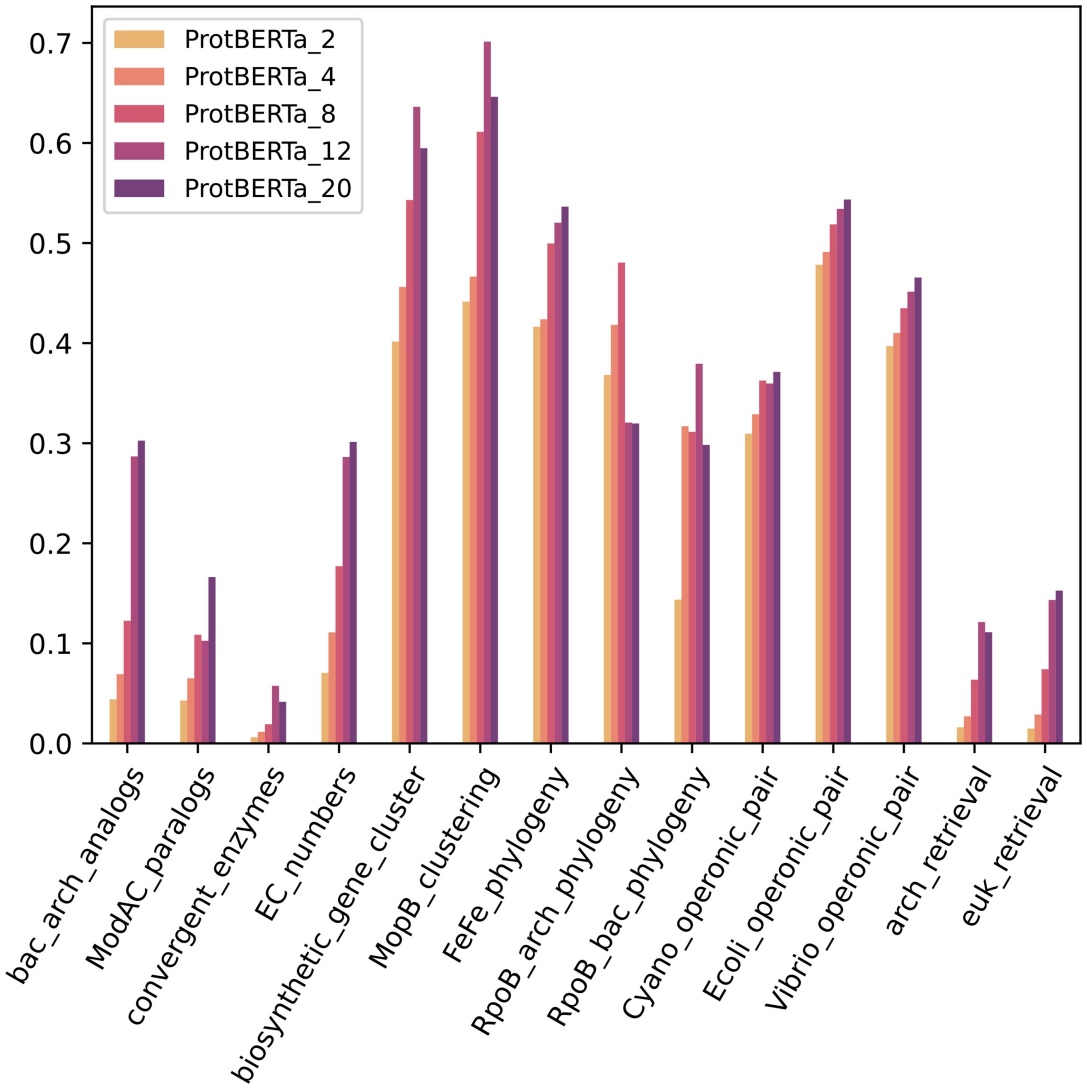
The performance of the different ProtBERTa models on primary metrics of the DGEB benchmark. The metrics descriptions are detailed in Table 2, available as supplementary data at *Bioinformatics* online.

We thus trained a BPE tokenizer (Sennrich, Haddow and Birch 2016) for each alphabet, resulting in different lengths of tokens (Table 2, Fig. 1A; Fig. 1, available as supplementary data at *Bioinformatics* online) and sentences (Fig. 1B, Fig. 2 and Table 1, available as supplementary data at *Bioinformatics* online). As the alphabet size decreases, recurring sequence patterns become more frequent, allowing BPE to merge characters into longer tokens. Thus, smaller alphabets yield more effective sequence compression and allow models to capture a broader context. This reduces running time and memory consumption, allowing utilization of more complex models (Vaswani et al. 2017; Korthikanti et al. 2023). We pre-trained RoBERTa, a transformer-based language model (Liu et al. 2019), on a large corpus of microbial proteins from genomic and metagenomic samples using each tokenizer. The models were called ProtBERTa_X, where X denotes the relevant alphabet size (2, 4, 8, 12, or 20).

**Table 2:** The ten most frequent tokens of each ProtBERTa (PBa) model observed in the tokenizer evaluation set.

Rank	PBa_20	PBa_12	PBa_8	PBa_4	PBa_2
1	LS	W	BB	N	B
2	AS	LN	SF	SS	BLLBBL
3	LI	LK	SSC	HNNN	BLBBBBL
4	LT	LL	AA	PHN	BBBBBBL
5	LD	AF	F	PPNN	BBLBBBL
6	LG	C	BH	HNNNN	BBLLBBL
7	LV	NK	SSM	SHN	LLBLBL
8	LE	LI	AH	PNNNN	BLBBBL
9	LK	KK	AC	SNNN	LLLBBL
10	AT	LE	FH	HHN	BBLLBL

### 3.2 Embedding quality assessment

#### 3.2.1 Evaluation on the diverse genomic embedding benchmark (DGEB)

To investigate the biological signals encoded in the embedding of each ProtBERTa model, we utilized the Diverse Genomic Embedding Benchmark (DGEB) (West-Roberts et al. 2024), a predefined embedding benchmark for biological language models. DGEB includes six different tasks across 18 curated datasets, spanning sequences from all domains of life. The benchmark comprises both nucleic acid and amino acid modalities, but given the nature of our tokenizers, we have only utilized the amino acid benchmarking tasks (detailed in Table 2, available as supplementary data at *Bioinformatics* online). This benchmark is based on the model’s pre-trained embeddings directly.

The best overall DGEB benchmark score, which aggregates performance across all tasks, was achieved by ProtBERTa_12 (DGEB score of 0.35), followed closely by ProtBERTa_20 (0.347) and ProtBERTa_8 (0.309, Table 2). While ProtBERTa_12 achieved the highest aggregate score, ProtBERTa_20 was the best-performing model for the majority of individual tasks (eight out of 14, Fig. 2). In contrast, ProtBERTa_2 and ProtBERTa_4 were not the top models for any individual DGEB task.

#### 3.2.2 Zero-shot homology prediction (pairwise binary classification)

**Figure 3:**
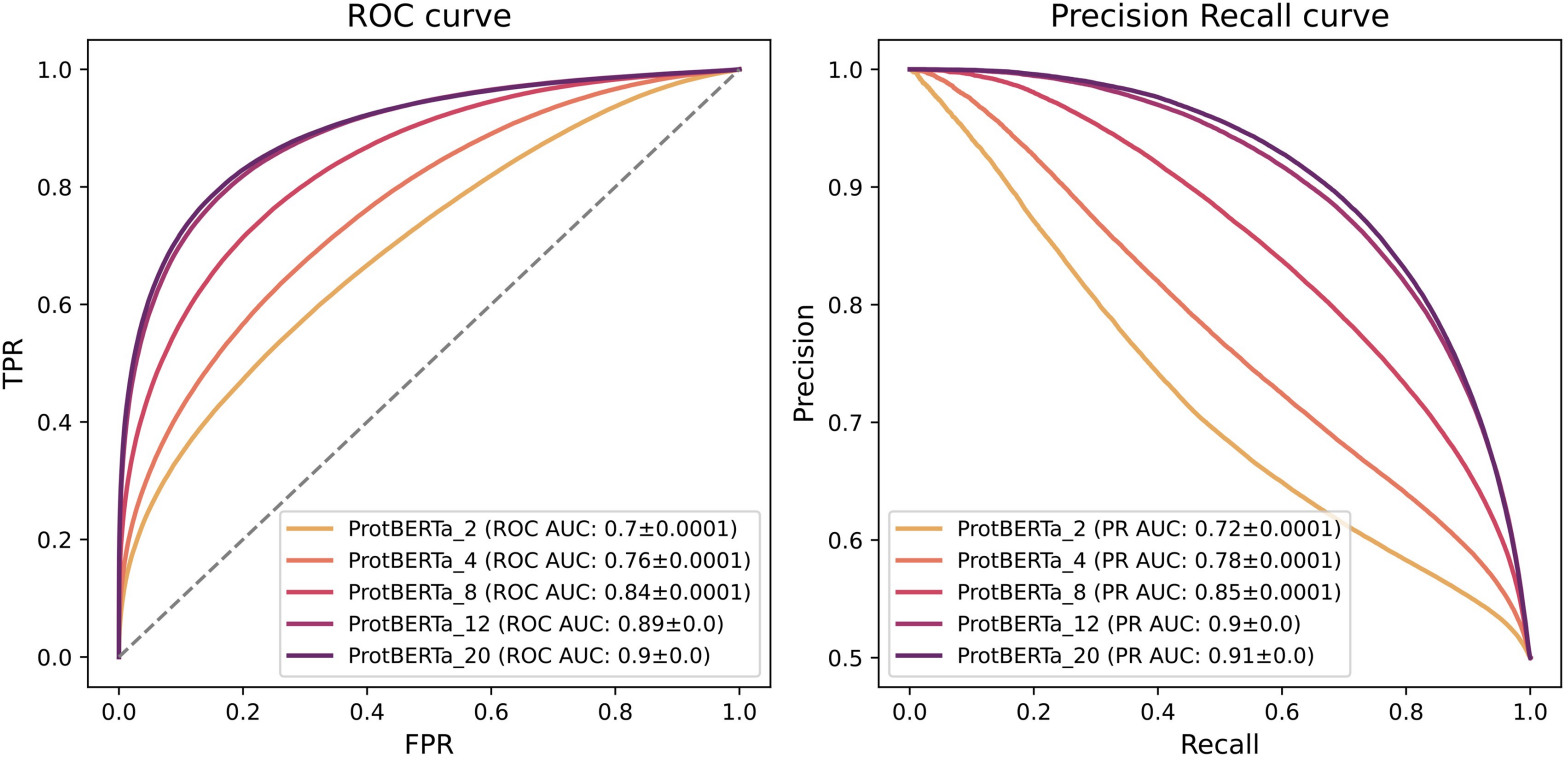
Zero-shot performance of the five ProtBERTa models on the task of homology detection. Left: ROC curves; right: precision–recall curves. The area under the curve score ± the standard error is noted in parentheses.

We further evaluated the signals encoded in the models’ embeddings by zero-shot homology prediction, where the cosine similarity of the pre-trained embeddings of pairs of proteins was used to train a model determining whether the two proteins belong to the same protein family. Even using raw embedding similarity alone, without any task-specific components, the classifiers based on the models’ embedding performed well (Fig. 3), with ProtBERTa_20 and ProtBERTa_12 achieving F1 scores of 0.818 and 0.813, respectively (Table 3, available as supplementary data at *Bioinformatics* online).

#### 3.2.3 Signal peptide detection (*k*-nearest neighbors)

**Figure 4:**
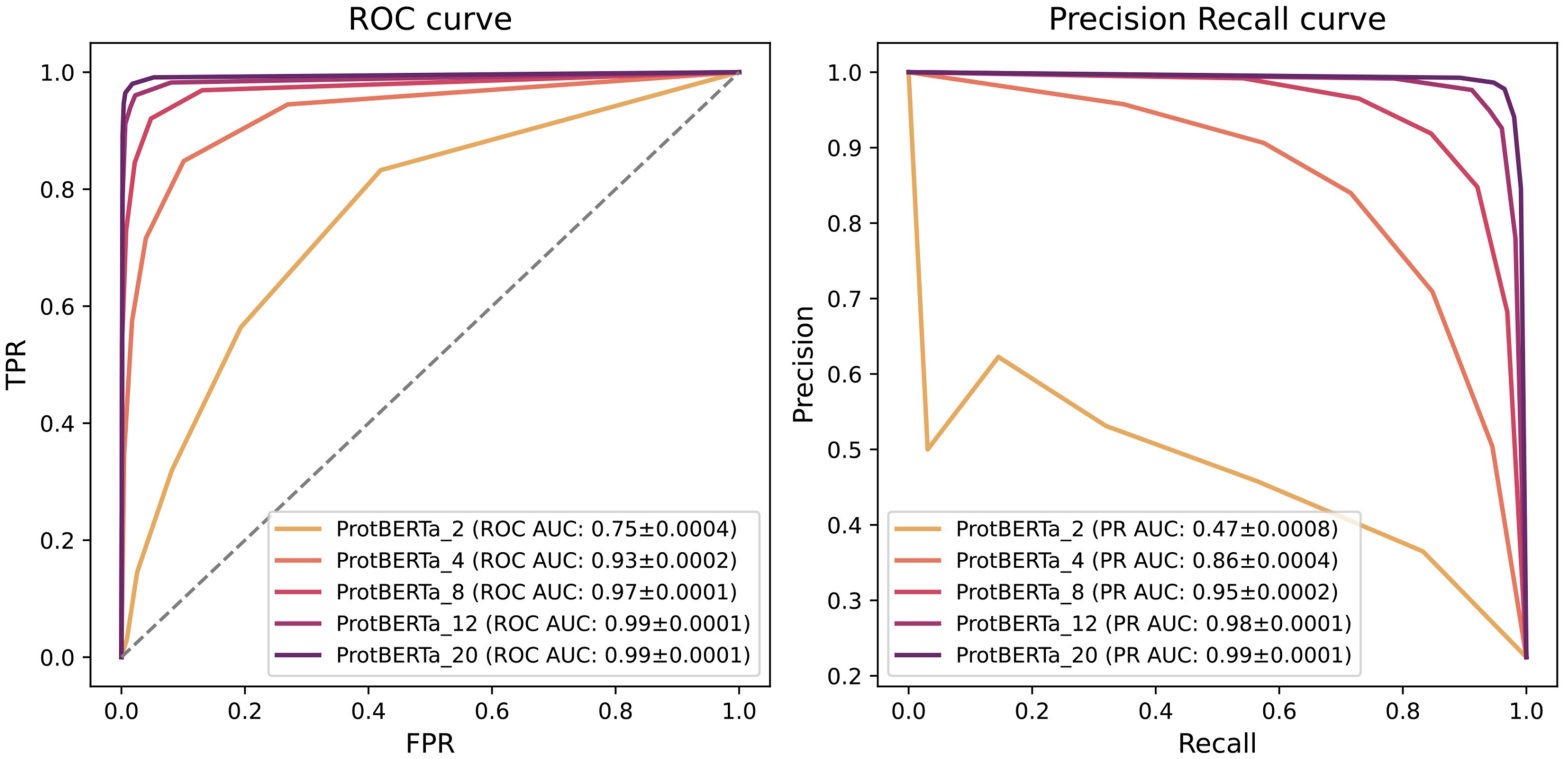
Performance of kNN classifiers using ProtBERTa pre-trained embeddings on the task of signal peptide classification. Left: ROC curves; right: precision–recall curves. The area under the curve score ± the standard error is written in parentheses.

We further assessed the models’ embeddings by training a *k*-nearest neighbors (kNN) classifier to identify signal peptides based on cosine similarity between the pre-trained embeddings. The labels of the test proteins were determined by majority vote among the *k *= 5 nearest train embeddings. ProtBERTa_20 achieved the best performance, with ProtBERTa_12 performing comparably (Fig. 4), while providing 1.28× compression (Fig. 3, available as supplementary data at *Bioinformatics* online).

### 3.3 Downstream task evaluation

#### 3.3.1 Classification tasks

**Figure 5:**
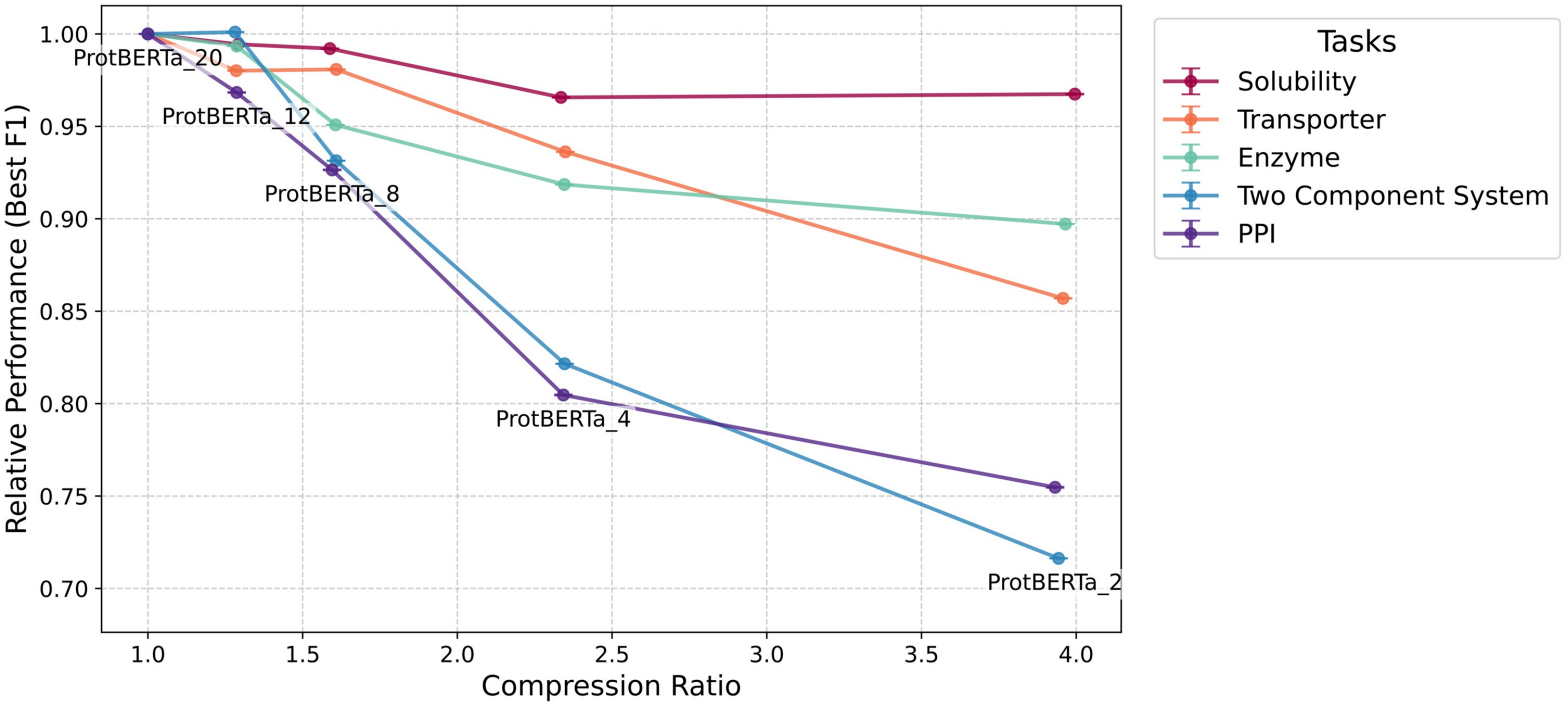
Performance versus sentence length compression trade-off of the different ProtBERTa models. The compression is calculated as average_sentence_length(ProtBERTa_x)/average_sentence_length(ProtBERTa_20), while the relative performance is calculated as best_f1(ProtBERTa_x)/best_f1(ProtBERTa_20) for the binary classification models, and macro_f1(ProtBERTa_x)/macro_f1(ProtBERTa_20) for the two component classification task. The relative weighted F1 of the two-component system task is shown in Fig. 9, available as supplementary data at *Bioinformatics* online.

The ProtBERTa models were further adapted for a variety of downstream classification tasks. Specifically, task-specific classification heads were trained to predict protein solubility, identify enzymes, transporters, and two-component systems, and detect protein-protein interactions (PPIs). While most models performed well across the tasks, ProtBERTa_20 consistently exhibited the highest classification performance, while ProtBERTa_12 and ProtBERTa_8 tended to achieve comparable performance (Fig. 5). In the solubility prediction task, where proteins were predicted as either soluble or membrane-bound, all models performed remarkably well, with ProtBERTa_8 achieving a slightly higher AUROC and AUPR (Fig. 4 and Table 4, available as supplementary data at *Bioinformatics* online), and ProtBERTa_20 marginally outperforming others in maximal F1. The performances of ProtBERTa_12 and ProtBERTa_8 were not statistically different from the performance of ProtBERTa_20 (*P*-values of .057 and .175, respectively, McNemar’s test), while ProtBERTa_8 achieved over 1.5× input compression (Fig. 5).

**Table 4:** The relative task-specific training time of all the ProtBERTa models (PBa) compared to the baseline model, PBa_20.

Task	PBa_12	PBa_8	PBa_4	PBa_2
Solubility	0.8 (0.77)	0.69 (0.63)	0.51 (0.43)	0.33 (0.25)
Enzyme	0.81 (0.78)	0.67 (0.62)	0.49 (0.43)	0.32 (0.25)
Transporter	0.78 (0.78)	0.65 (0.62)	0.47 (0.43)	0.32 (0.25)
TCS	0.79 (0.78)	0.66 (0.62)	0.47 (0.43)	0.32 (0.25)
PPI	0.79 (0.78)	0.66 (0.63)	0.46 (0.43)	0.29 (0.25)
Stability	0.96 (0.8)	0.94 (0.69)	0.89 (0.52)	0.83 (0.35)
Temperature	0.78 (0.78)	0.64 (0.63)	0.43 (0.43)	0.25 (0.25)
Fluorescence	0.86 (0.76)	0.79 (0.62)	0.63 (0.42)	0.55 (0.24)

Values in parentheses indicate the relative input size (1/compression_ratio), which correlates with the runtime.

Abbreviation: TCS = two-component systems.

**Figure 6:**
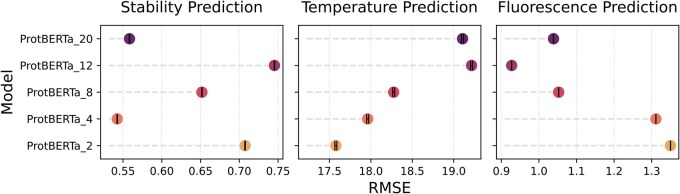
RMSE scores of the five ProtBERTa models on the regression tasks. Left: Protein stability prediction. Middle: Optimal protein temperature prediction. Right: Fluorescence prediction. Error bars represent standard error.

**Figure 7:**
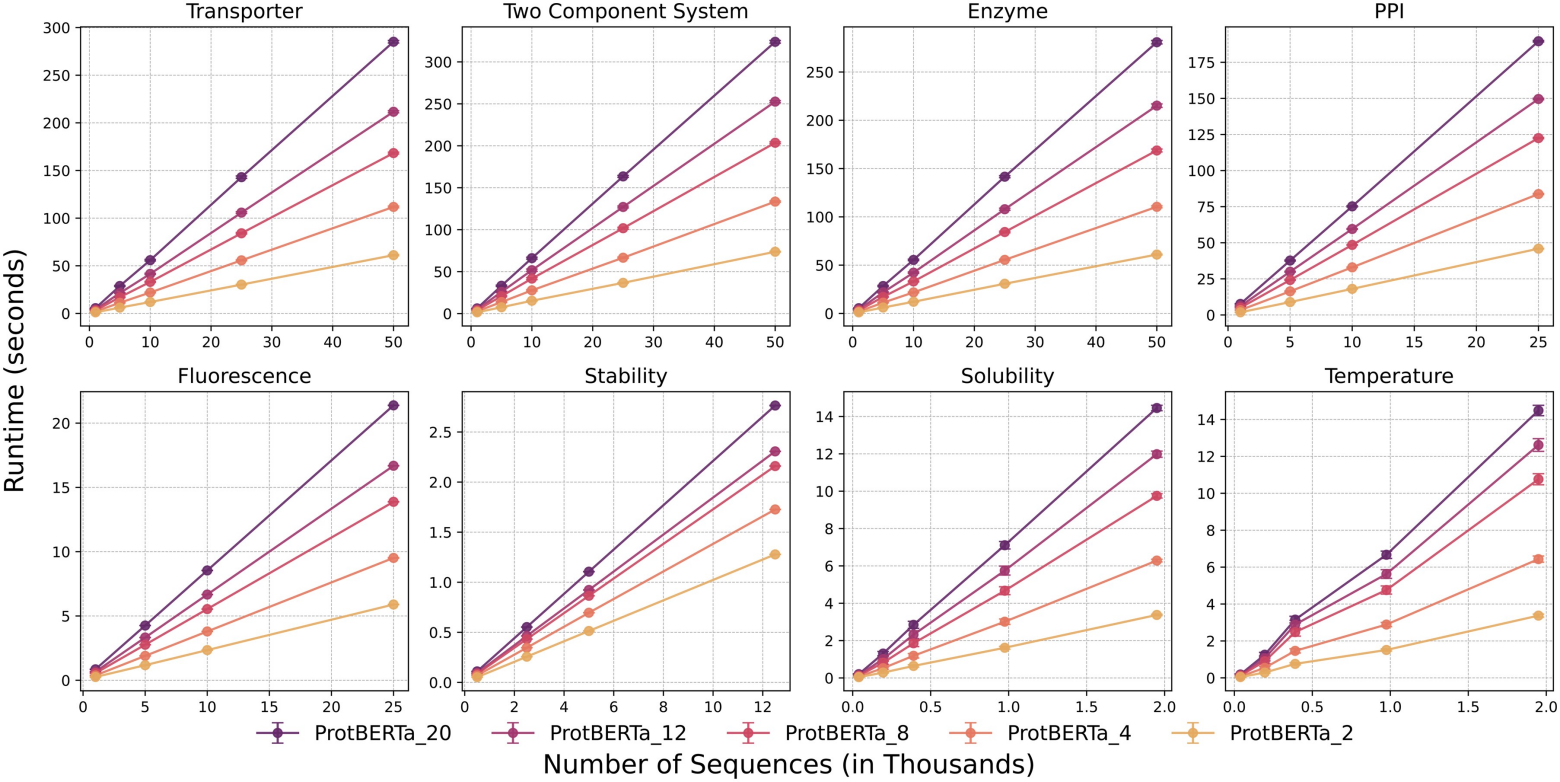
Inference run time comparison for the five ProtBERTa models on a variety of downstream tasks. Inference time is measured as a function of the dataset size (in thousands). Error bars represent standard error.

For the enzyme detection task, ProtBERTa_20 and ProtBERTa_12 consistently achieved the strongest overall performance (Fig. 5 and Table 5, available as supplementary data at *Bioinformatics* online). For the transporter task, ProtBERTa_20, ProtBERTa_12, and ProtBERTa_8 achieved comparable performance (Fig. 6 and Table 6, available as supplementary data at *Bioinformatics* online). In both cases, ProtBERTa_8 provided over 1.5× input compression with only modest performance degradation (2.5–5.5%, Fig. 5). In the two-component system multiclass task, where each protein is assigned as either a sensor, a response regulator, or neither, ProtBERTa_12 performed comparably to ProtBERTa_20 (*P*-value of .354, McNemar’s test) with over 1.28× compression (Fig. 5, Fig. 7 and Table 7, available as supplementary data at *Bioinformatics* online). In contrast, the protein–protein interaction task showed a clearer dependence on alphabet size, with performance decreasing as the alphabet was reduced (Fig. 8 and Table 8, available as supplementary data at *Bioinformatics* online). Nevertheless, ProtBERTa_8 still provided over 1.5× input compression with relatively limited performance loss (93% of ProtBERTa_20’s AUPR and best F1, Fig. 5).

#### 3.3.2 Regression tasks

We also explored the performance of different ProtBERTa models on regression tasks, predicting protein stability, optimal temperature, and fluorescence levels. On the protein stability prediction task, the performance improved as the alphabet size increased, with ProtBERTa_4 demonstrating the best performance with an RMSE score of 0.543, and ProtBERTa_20 following, with an RMSE score of 0.558 (Fig. 6A, Table 9, available as supplementary data at *Bioinformatics* online). Conversely, for the optimal temperature prediction task, model performance improves as the alphabet size decreases, with ProtBERTa_2 outperforming the other models with an RMSE score of 17.577 (Fig. 6B, Table 10, available as supplementary data at *Bioinformatics* online). Finally, on the fluorescence prediction task, ProtBERTa_12 outperformed all the other models, achieving an RMSE score of 0.927 (Fig. 6C, Table 11, available as supplementary data at *Bioinformatics* online). While ProtBERTa_20 and ProtBERTa_8 also performed relatively well.

### 3.4 Runtime comparison

Since the input sequence length directly affects computational cost, we hypothesized that the input compression achieved by reduced-alphabet tokenization would translate to shorter training and inference time. To verify this, we compared the running time of the models on downstream biological tasks described above. We observed that the decreases in training time roughly followed the models’ compression ratios. Specifically, ProtBERTa_4 required approximately half the training time of ProtBERTa_20, while ProtBERTa_2 required roughly one third (Table 3). We observed a similar trend for the tokenizer training time (Fig. 10, available as supplementary data at *Bioinformatics* online). In contrast, tokenization times were nearly identical across ProtBERTa_20, ProtBERTa_12, and ProtBERTa_8, with ProtBERTa_2 having slightly lower tokenization times (Fig. 11, available as supplementary data at *Bioinformatics* online).

**Table 3:** DGEB scores of the different ProtBERTa (PBa) models. The best performance is marked in bold.

Model	PBa_2	PBa_4	PBa_8	PBa_12	PBa_20
DGEB Score	0.225	0.259	0.309	**0.35**	0.347

This trend is consistent with the computational structure of transformer models, which comprise self-attention layers with $O(s^{2}d)$ complexity and feed-forward layers with $O(sd^{2})$ complexity, where *s* is the sequence length and *d* is the hidden dimension size. Since most tokenized sequences in our datasets are shorter than 200 tokens, with a mean length below 123 tokens (Fig. 1B, Table 1, available as supplementary data at *Bioinformatics* online), and the models’ hidden dimension size is 768, d has a greater contribution to the overall computation. Consequently, the feed-forward layers are expected to have a greater impact on runtime than the self-attention layers. This may explain the approximately linear scaling of runtime with input length, and the observed reductions in training time.

We note that, except for the PPI model, which was fully fine-tuned, all encoder parameters were frozen, with only the task-specific heads updated. In addition, the stability prediction dataset contains unusually short proteins (mean length of 43.2 amino acids), which likely accounts for the smaller-than-expected runtime differences observed for this task relative to others.

We also compared the inference time of the models according to the test set sample size. We observed that a similar trend also occurred during the inference stage, where the models exhibited approximately the same runtime ratio across different sizes of datasets, with ProtBERTa_20 running about four times longer than ProtBERTa_2. A linear relationship between the runtime and number of sequences was observed across the different tasks and models (Fig. 7).

## 4 Conclusion

Although reduced amino acid alphabets have been widely used in bioinformatics (Murphy et al. 2000, Weathers et al. 2004, Peterson et al. 2009, Ofer and Linial 2015) and previously evaluated in the context of protein language models (Ieremie et al. 2024), they had not been combined with sub-word tokenization to improve pLM efficiency. Our results indicate that, while the full 20-amino-acid alphabet achieves the highest performance on most tasks, the models trained on the reduced alphabets have outperformed the baseline model on specific tasks. Notably, models trained on reduced alphabets typically incurred only minor performance loss, while providing substantial reductions in training and inference times.

We hypothesize that the decreased performance of models trained with reduced alphabets on classification tasks stems from the loss of fine-grained biochemical information encoded by specific amino acids. This effect is particularly pronounced in the protein–protein interaction (PPI) prediction task, where precise residue identities are critical for mediating physical interactions. In contrast, for optimal temperature prediction, performance improves as the alphabet size decreases. This may reflect the relatively small dataset combined with high label variability, making the task inherently challenging; under such conditions, learning more generalized representations encoding global thermodynamic signatures that drive thermal stability, while filtering out sequence-specific noise that can lead to overfitting, could be advantageous. The fluorescence and stability prediction tasks represent a middle ground, where an intermediate alphabet (12 characters for fluorescence, 4 for stability) performs best, possibly because it captures the necessary evolutionary constraints of the protein fold while maintaining enough relevant chemical details. Nevertheless, our findings do not allow for a definitive explanation of why certain alphabet sizes are better suited to specific tasks. We therefore recommend evaluating multiple alphabet configurations when applying subword tokenization-based protein language models, as reduced alphabets can outperform standard representations in certain settings, while in others they achieve comparable performance with the added benefit of reduced computational cost.

We note that while a full-alphabet model could, in principle, learn similar generalizations from sufficient labeled training data, in practice, this may be difficult to achieve. A model pre-trained extensively on the full 20-amino acid alphabet develops rich, fine-grained representations that distinguish between all amino acids. When applied to a downstream task with a small dataset, such distinctions are noise rather than signal; the model must effectively unlearn these pre-trained representations, a process that requires substantial data and may not converge when limited labeled data are available. A reduced-alphabet model, by contrast, never encodes these distinctions in the first place, providing representations that are already at the appropriate resolution for the task. This is analogous to the choice of amino acids over codons as the basic unit of protein modeling.

To ensure a fair comparison, all models were pre-trained and applied to downstream tasks using identical hyperparameters. While task-specific and model-specific hyperparameter optimization could alter performance trends and potentially reduce differences between models, we avoided this approach to better isolate the effects of alphabet reduction and the associated physicochemical properties. Future work could explore model-specific optimization strategies as well as additional reduced alphabets.

It is worth noting that our study focuses on the impact of integrating reduced amino acid alphabets within subword tokenization for pLMs. Accordingly, our primary baseline is a 20-amino acid BPE tokenizer, rather than the character-level pLMs commonly used in prior work. Character-level models are typically more appropriate for residue-level biological tasks, for which BPE-based tokenization is less suitable. Reduced alphabets may also obscure mutations between amino acids within the same group, limiting applications such as variant effect prediction. These tasks would therefore benefit from alternative tokenization strategies.

A natural direction for future work is a systematic comparison between reduced-alphabet BPE models, character-level baselines, and existing pLMs in terms of performance and efficiency. Additionally, comparing our approach against alternative efficiency strategies, such as knowledge distillation or smaller architectures, would be valuable.

Furthermore, our experiments were conducted using a relatively small RoBERTa-based pLM. We expect similar trends to hold for larger models, particularly with respect to speed improvements in attention-based architectures, as the degree of sequence compression is determined by the tokenizer. However, further investigation is needed to assess the effects of reduced alphabets and BPE tokenization across models of varying architectures and scales.

Overall, our findings highlight the merits of combining reduced amino acid alphabets with sub-word tokenization as an effective strategy for compressing pLM input sequences, enabling more efficient training and inference while preserving biologically meaningful signals.

## Supplementary Material

btag226_Supplementary_Data

## Data Availability

The datasets used in this study were obtained from publicly available sources, including EMBL-EBI MGnify (https://www.ebi.ac.uk/metagenomics) and NCBI Whole Genome Shotgun (WGS) (https://www.ncbi.nlm.nih.gov/genbank/wgs). The code supporting this work is publicly available on GitHub at https://github.com/burstein-lab/BioTokenizers. The trained tokenizers and pre-trained models are available via Zenodo at https://doi.org/10.5281/zenodo.18256943 and https://doi.org/10.5281/zenodo.18257091.
